# The effect of exercise intervention on improving sleep in menopausal women: a systematic review and meta-analysis

**DOI:** 10.3389/fmed.2023.1092294

**Published:** 2023-04-25

**Authors:** Jialu Qian, Shiwen Sun, Man Wang, Yaping Sun, Xiangyu Sun, Cecilia Jevitt, Xiaoyan Yu

**Affiliations:** ^1^Department of Obstetrics, Women's Hospital School of Medicine, Zhejiang University, Hangzhou, China; ^2^Department of Family Medicine, Faculty of Medicine, University of British Columbia, Vancouver, BC, Canada

**Keywords:** exercise intervention, menopause, sleep quality, insomnia, meta-analysis

## Abstract

**Background:**

Sleep disturbance is common in menopausal women and negatively affects their quality of life and could cause increased risks of other menopause-related diseases.

**Objective:**

This systematic review aims to synthesize evidence regarding the effects of exercise interventions on improving sleep in menopausal women.

**Methods:**

A comprehensive search in seven electronic databases for randomized controlled trials (RCTs) was performed on June 3, 2022. The systematic review included seventeen trials, ten of which provided data for the meta-analysis. The effects on outcomes were presented as mean differences (MDs) or standard mean differences (SMDs) and their 95% confidence intervals (CI). Cochrane risk-of-bias tool was used in quality assessment.

**Results:**

The results suggest that exercise intervention significantly reduces insomnia severity (SMD = −0.91, 95% CI = −1.45 to −0.36, *Z* = 3.27, *P* = 0.001) and alleviates sleep problems (MD = −0.09, 95% CI = −0.17 to −0.01, *Z* = 2.20, *P* = 0.03). For sleep quality, the results showed that insignificant differences were found between the exercise intervention and the control groups (MD = −0.93, 95% CI = −2.73 to 0.87, Z = 1.01, *P* = 0.31). The results of the subgroup analysis indicated that more apparent effects of exercise intervention were found among women with sleep disorders than among women without sleep disorders. Which exercise intervention duration was more beneficial to sleep outcomes could not be judged. Overall, there was a moderate risk of bias in the primary studies.

**Conclusion:**

According to this meta-analysis, exercise interventions can be recommended for menopausal women to improve their sleep. High-quality RCTs applying different types of exercise (e.g., walking, yoga, meditative exercise and so on) with different intervention durations as well as subjective and objective sleep assessment are warranted.

**Systematic review registration:**

https://www.crd.york.ac.uk/prospero/display_record.php?ID=CRD42022342277, identifier: CRD42022342277.

## 1. Introduction

Menopause is the phase in which reproductive life ends and senescence starts which occurs at ~50 years of age. Women currently spend approximately one-third of their lives with menopause (1). More women experience this period because of increased life expectancy (2). Menopause is a biopsychosocial turning point for women's health (3); however, it is frequently accompanied by bothersome symptoms, including vasomotor symptoms, mood disturbances, temporarily impaired cognitive function, genitourinary complaints, and other disease processes that can negatively affect menopausal women's sleep (4).

Sleep disturbance is increasingly recognized as a major problem in women's wellbeing, particularly in the context of menopause (5). Sleep disturbances are associated with the menopausal transition. Menopausal women have reported insomnia as one of their most common symptoms (6, 7). A 10-year multi-ethnic study found that 46–48% of menopausal women and 38% of premenopausal women experienced insomnia (8). Sleep problems occurring during the menopausal stage may lead to an increased risk of arterial stiffness, hypertension, diabetes, and cardiovascular disease (9–11). Research indicates that midlife women may suffer long-term mental and physical health problems from untreated insomnia, in addition to immediate changes in health care usage and quality of life (12).

Women's healthcare needs at menopause have been under recognized and underserved by health care professionals for a long time (3). Considering the high prevalence rates and adverse consequences of sleep problems timely recognition of menopausal sleep problems and effective strategies to relieve sleep disturbance symptoms are needed (4).

Women who experience menopausal symptoms are often treated with hormone therapy. An increased risk of cardiovascular disease, breast cancer, gallstones and dementia was found in menopausal women who received hormone therapy (13, 14). The health risks associated with menopausal hormone therapy have led patients and clinicians to question its safety (15). Therefore, many women are seeking relief from menopause-related symptoms through complementary and alternative medicine. Exercise intervention, as one of the available non-pharmacological therapies, can offer health benefits without the limitations of hormone therapy. Exercise intervention is safe and has no obvious side effects. The effectiveness of exercise on improving sleep quality and decreasing insomnia has been documented among different populations (16, 17). One study explored possible mechanisms of the beneficial effects of exercise on sleep deprivation (18).

In the field of menopause management, exercise intervention has been associated with effectively alleviating sleep problems (19–21); however, exercise has not been shown to significantly improve sleep in some studies (22, 23). A previous meta-analysis of four RCTs in 2016 investigated the effects of exercise on sleep quality and insomnia in women of middle age (24). However, limited subgroup analysis was conducted because of small number of included studies. Meanwhile, studies of exercise interventions implemented in menopausal women to improve their sleep have accumulated during the past 5 years (21, 25–28). The objective of this systematic review was to provide the latest evidence of the effectiveness of exercise intervention on improving sleep in menopausal women. We also conducted further subgroup analysis to explore whether the effect sizes for sleep quality and severity of insomnia changed based on the intervention population (with or without sleep disorders) and the intervention duration (≤3 months or >3 months) to provide more evidence in this field.

## 2. Method

### 2.1. Protocol and registration

This systematic review and meta-analysis was conducted in accordance with the Preferred Reporting Items for Systematic review and Meta-Analysis (PRISMA) extension guidelines (29). It was preregistered in the International Prospective Register of Systematic Reviews (PROSPERO) (30) (registration: CRD42022342277).

### 2.2. Search strategy

A comprehensive search of the literature was performed in PubMed, Embase, Cochrane Central Register, CINAHL, ProQuest, Scopus, and Web of Science to identify articles that examined the effects of exercise intervention on sleep improvement. Combinations of Medical subject headings (MeSH) and keywords were utilized. The detailed search strategies are summarized in Supplementary Table 1. The search was performed independently by two authors (JLQ, SWS) for the period from inception until 3 June 2022. A backwards search was conducted of reference lists that included articles and relevant systematic reviews until no additional relevant articles were found.

### 2.3. Study selection

The inclusion criteria were assessed using the PICOS approach. (1) Population: premenopausal, menopausal and postmenopausal women. (2) Intervention: potential exercise interventions might include but were not limited to the following: yoga, walking or aerobic exercise. There was no restriction on the setting, frequency, or duration of the intervention. (3) Comparison: waiting list condition, usual care or no intervention. (4) Outcome: studies that reported sleep-related outcomes, such as the Pittsburgh Sleep Quality Index (PSQI), Insomnia Severity Index (ISI) and sleep diary measures (total sleep time, wake time after sleep onset, long awakenings, sleep onset latency, wake time after sleep onset, sleep efficiency). PSQI is a validated sleep assessment tool that evaluates sleep disturbances and sleep quality over 1 month. ISI measures sleep quality over the past 2 weeks. The established cut-offs of 5 points on the PSQI (31) and 10 points on the ISI mean score (32) were used to indicate clinical levels of insomnia. (5) Study design: randomized controlled trials (RCTs). Criteria for exclusion were as follows: (1) women with musculoskeletal disorders, heart disease, arterial hypertension, diabetes, and cancer or women who received hormone replacement therapy; (2) studies carrying out health education intervention aimed at improving the physical activity of menopausal women; (3) studies integrating exercise interventions with pharmacological interventions; and (4) duplicated publications.

We imported identified references into EndNote X9 software to manage data more efficiently. First, we excluded irrelevant studies by removing duplicates and screening titles and abstracts independently by two authors (JLQ, SWS). Then, evaluation was conducted on the full texts of the remaining references based on the inclusion and exclusion criteria. Third, three authors (JLQ, SWS, MW) discussed the studies to reach a consensus on the inclusion of studies if any discrepancy appeared.

### 2.4. Data extraction

A standardized form was adopted for extracting data from eligible studies. The following information were extracted: first author, country, year of publication, participants, sample size, type of exercise, intervention frequency, session, duration, control, evaluation time points, and assessment tools. Two authors (JLQ and SWS) cross-checked the accuracy of data extracted from each study. An independent third author (MW) resolved any inconsistencies.

### 2.5. Data analysis

Review Manager (RevMan) version 5.3 was adopted to conduct all data analyses. Mean differences (MDs) and 95% confidence intervals (CIs) for all individual sleep outcomes (number of studies ≥ 2) were calculated separately if the outcomes were measured choosing the same tool. We combined studies using the standard mean difference (SMD) and 95% confidence intervals (CI) when the same outcome was measured by different methods (33, 34). According to the SMD, effect sizes of 0.2, 0.5, and 0.80 are considered small, moderate, and large, respectively (35). Between-study heterogeneity was examined by standard chi-square and quantified with *I*^2^ statistics (36). If the *P*-value was >0.1 or *I*^2^ <50%, and then a fixed effect model was conducted. Otherwise, the researchers used a random-effect model analysis (37). A subgroup analysis was also carried out based on the intervention population (with or without sleep disorders) and the intervention duration (≤3 months or >3 months). Egger's test was used to assess publication bias.

### 2.6. Quality assessment

A Cochrane risk-of-bias tool was utilized by two independent authors (JLQ and SWS) to assess the risk of bias (38). Seven dimensions are outlined in the tool for estimating the risk of bias in each study. We assessed each domain individually to determine whether it posed a low risk, high risk, or unclear risk of bias. An interrater reliability score using Cronbach's alpha for risk of bias was used for the original grading done by two raters.

## 3. Results

### 3.1. Literature search

**Figure 1:**
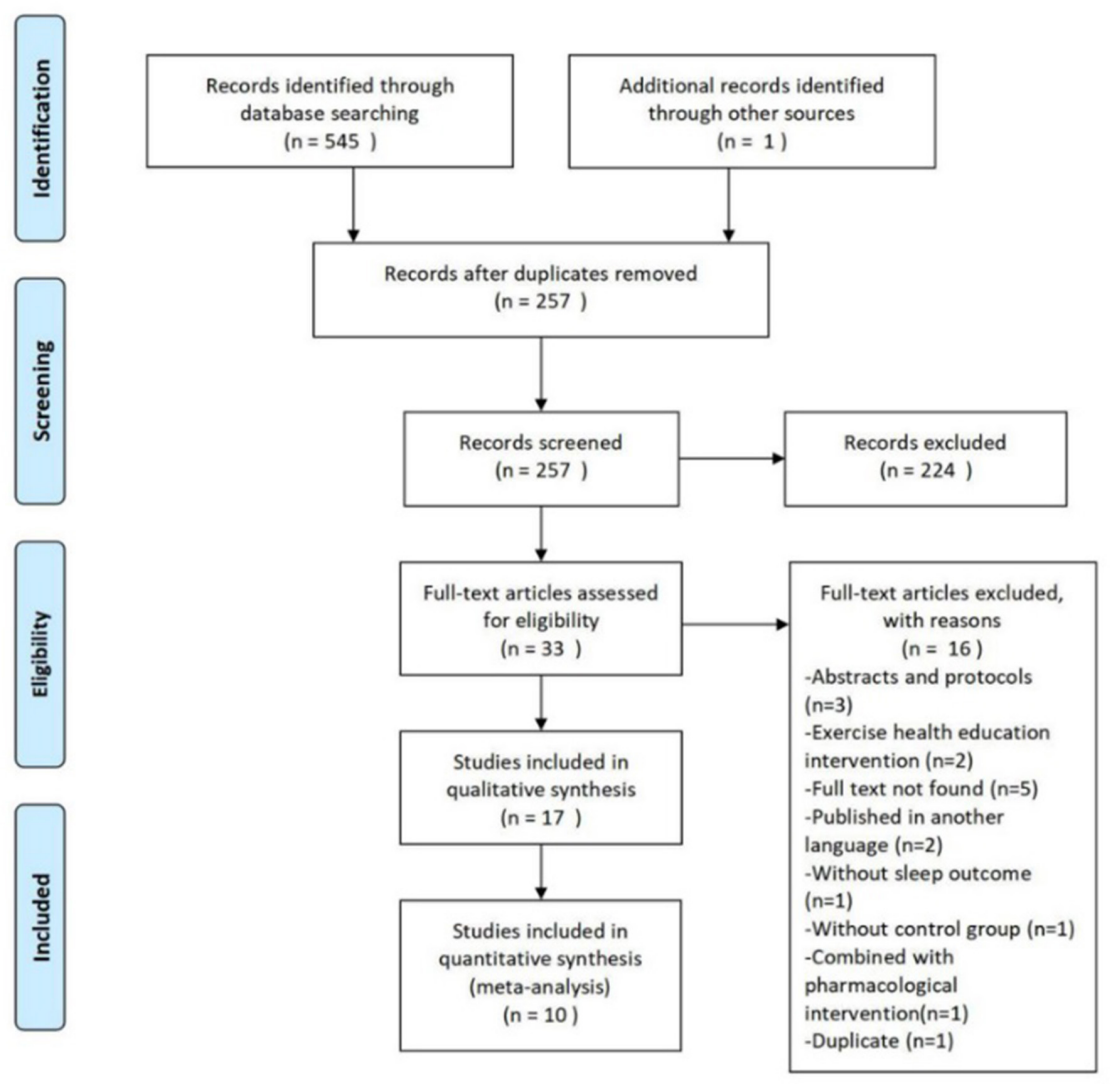
PRISMA study inclusion flowchart.

An overview of the study selection process are summarized in Figure 1. The initial search of seven databases identified 545 citations. One other relevant study was found after searching reference lists. Following the removal of 289 duplicate articles, we screened the titles and abstracts of the remaining 257 articles. Full text papers were ordered for 33 citations. Seventeen studies were included for the systematic review, and ten of those provided statistics for meta-analysis.

### 3.2. Characteristics of included studies

The study characteristics are summarized in Supplementary Table 2. Studies were published between 2004 and 2022. A total of 17 articles from different countries were included in the review: the USA (*n* = 7), Iran (*n* = 3), China (*n* = 2), Finland (*n* = 2), Brazil (*n* = 1), Spain (*n* = 1) and Sweden (*n* = 1). This review involved 2,463 participants. Exercise intervention types were grouped into the following categories: walking (20, 22, 27, 39–41) (*n* = 6), yoga (22, 26, 42, 43) (*n* = 4), aerobic exercise (19, 26, 44, 45) (*n* = 4), passive stretching (27, 42) (*n* = 2), integrated-style exercise (23, 27) (*n* = 2), Pilates training (21) (*n* = 1), resistance training (25) (*n* = 1), treadmill exercise (46) (*n* = 1) and Tai Chi Chuan (28) (*n* = 1). The interventions durations ranged from 3 to 12 months.

### 3.3. Main results

**Figure 2:**
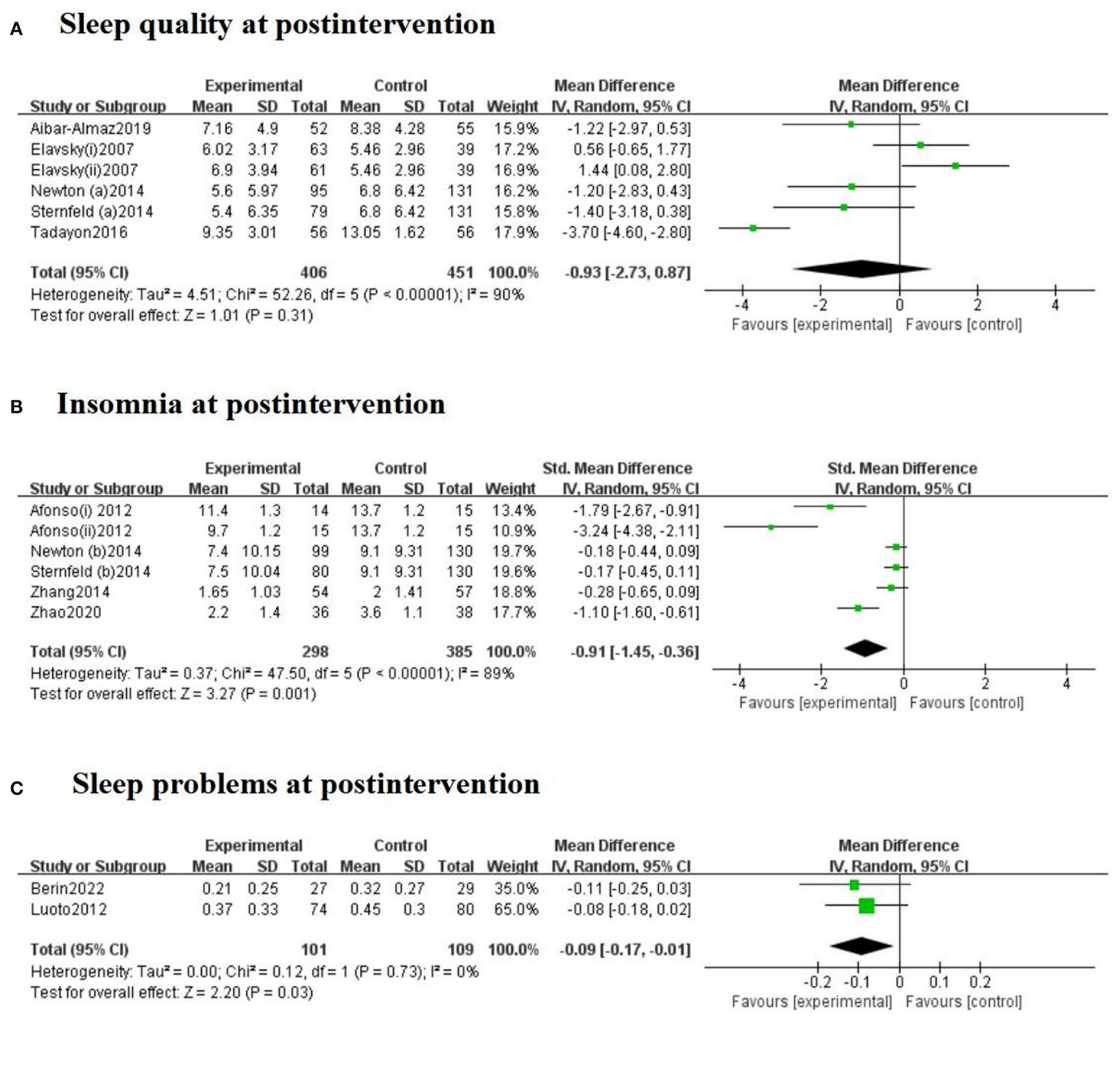
Forest plots for the effect of exercise training on sleep quality **(A)**, insomnia **(B)**, and sleep problems **(C)** at postintervention. The Afonso et al. (42) study included two intervention groups (i: passive stretching and ii: yoga), which had assessment scores. The Elavsky and McAuley (22) study included two intervention groups (i: walking and ii: yoga).

Meta-analyses revealed differences between exercise intervention and control groups when it came to sleep outcomes, including sleep quality, insomnia and sleep problems, in menopausal women, as displayed in Figure 2. There were five trials with PSQI post-assessment. A random-effects model was chosen due to the significangt heterogeneity (*I*^2^ = 90%). The pooled MD was −0.93 (95% CI = −2.73 to 0.87, *Z* = 1.01, *P* = 0.31), indicating that the exercise intervention had no significant effects on improving sleep quality. Exercise intervention led to significant reductions in insomnia symptoms in five RCTs among menopausal women (SMD = −0.91, 95% CI = −1.45 to −0.36, *Z* = 3.27, *P* = 0.001) compared with the control group. The heterogeneity was high (*I*^2^ = 89%), and therefore a random-effects model was applied. Regarding sleep problems measured by WHQ, two trials showed that exercise had favorable effects on the WHQ scores (MD = −0.09, 95% CI = −0.17 to −0.01, *Z* = 2.20, *P* = 0.03) compared to controls. As there was no significant heterogeneity, we used a fixed-effects model (*I*^2^ = 0%, *P* = 0.73).

### 3.4. Subgroup analysis

Subgroup analyses were performed to determine whether the effect sizes for sleep quality and severity of insomnia changed based on the intervention population (with or without sleep disorders) and the intervention duration (≤3 months or >3 months). For sleep quality, different intervention populations showed significant differences (see Supplementary Figure 1). Positive effects of exercise intervention on sleep quality improvement were found among menopausal women with sleep disorders (MD = −3.70, 95% CI = −4.60 to −2.80, *Z* = 8.10, *P* < 0.00001) in one study. However, exercise intervention appeared to be ineffective in menopausal women without sleep disorders (MD = −0.06, 95% CI = −0.73 to 0.61, *Z* = 0.17, *P* = 0.86) in five studies. For the severity of insomnia, two studies applying exercise intervention among women with sleep disorders (SMD = −2.47, 95% CI = −3.89 to −1.05, *Z* = 3.41, *P* = 0.0007) had a larger effect size than those without sleep disorders in four studies (SMD = −0.38, 95% CI = −0.72 to −0.04, *Z* = 2.22, *P* = 0.03).

Supplementary Figure 2 displays the forest plots of the subgroup analyses based on the intervention duration (≤3 months or >3 months). For sleep quality, different intervention durations result in significant differences. Four studies with ≤3 months of exercise intervention duration (MD = −0.20, 95% CI = −3.51 to −0.49, *Z* = 2.59, *P* = 0.005) appeared to be much more effective on PSQI score reduction than two studies with >3 months of intervention duration (MD = 0.95, 95% CI = 0.05 to 1.86, *Z* = 2.06, *P* = 0.31). For the severity of insomnia, three studies conducting >3 months of intervention (SMD = −1.95, 95% CI = −3.10 to −0.80, *Z* = 3.33, *P* = 0.0009) had a higher effect size than three studies with ≤3 months of intervention duration (SMD = −0.19, 95% CI = −0.36 to −0.02, *Z* = 2.23, *P* = 0.03).

### 3.5. Risk of bias

**Figure 3:**
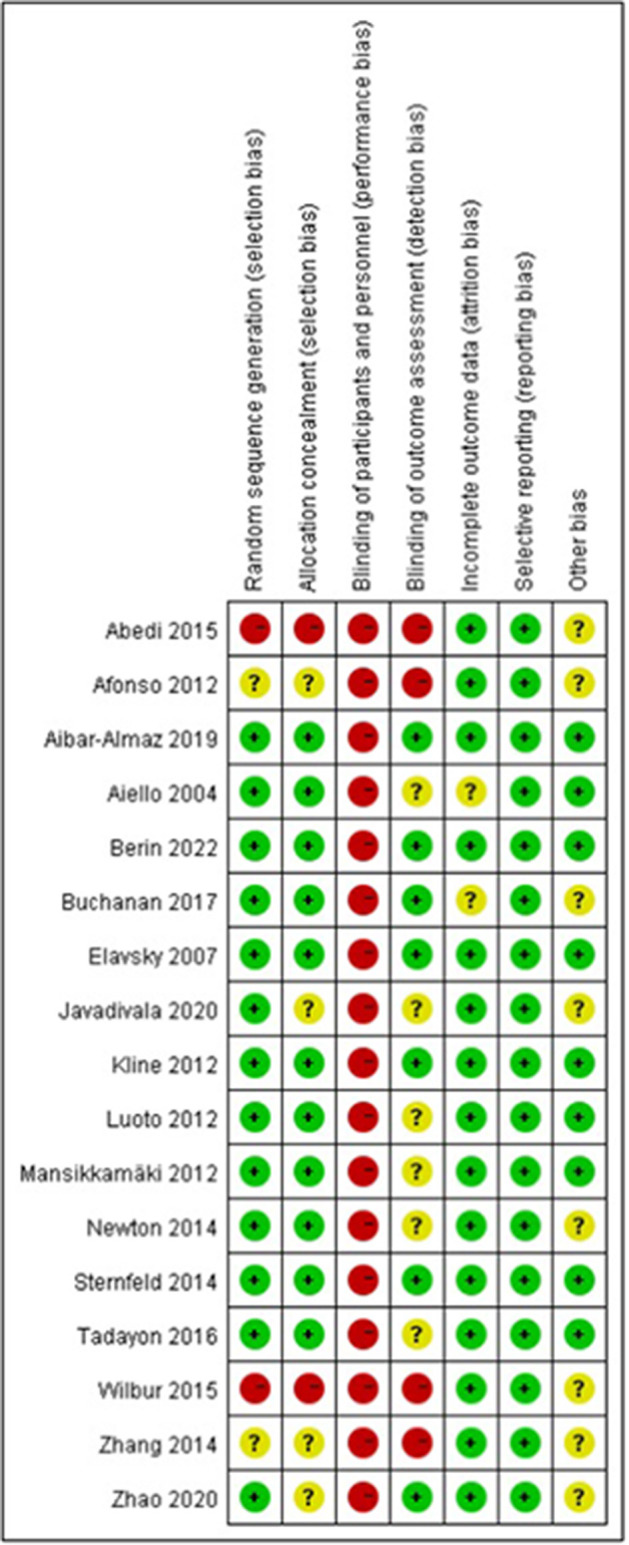
Risk of bias summaries for individual studies.

**Figure 4:**
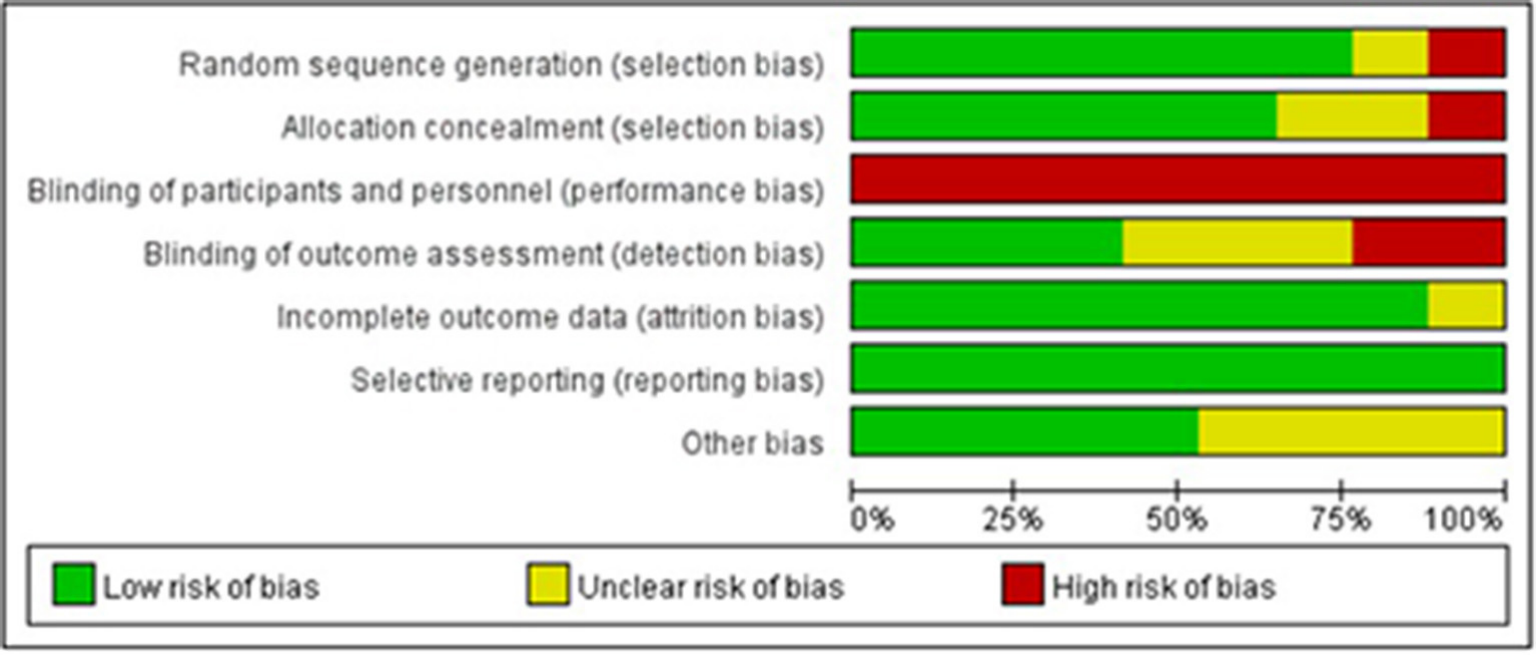
Risk of bias summaries for all studies.

Figures 3, 4 illustrate the results of the risk of bias assessment using the Cochrane tool. The majority of studies had detailed descriptions of the generation of randomization sequence, concealment of allocations, incomplete outcome data and selective outcome reporting processes, and the risk of bias was found to be low for most trials in terms of these aspects. The nature of the intervention made it impossible to blind study participants or researchers. Hence, performance bias is present in all of the studies. A total of seven trials reported blinding of outcome assessment (21, 22, 25, 26, 28, 45, 46). The Cronbach's alpha for the seven assessment dimensions ranged from 0.8 to 1.0.

### 3.6. Publication bias

Using funnel plots for <10 articles is inappropriate. Hence, Egger's tests were conducted for the outcomes sleep quality and insomnia. Egger's showed no evidence of publication bias for sleep quality (*t* = −0.3, *P* = 0.777). However, significant publication bias was observed with regard to insomnia (*t* = −7.76, *P* = 0.01).

## 4. Discussion

Despite the high prevalence and serious consequences of sleep problems during the menopausal period, well-conducted studies on exercise intervention for improving sleep among menopausal women are scarce. This systematic review identified seventeen trials exploring the effectiveness of exercise interventions on sleep outcomes in menopausal women, with ten studies providing statistics for meta-analyses. Statistically significant differences were observed in ISI and WHQ scores resulting from exercise interventions in menopausal women. However, at post-intervention, sleep quality was insignificantly different between the exercise intervention and control groups. Subgroup analysis indicated that the exercise intervention implemented among menopausal women with sleep disorders had better effects on sleep quality and insomnia. The influence of the intervention duration (≤3 months or >3 months) on exercise efficacy needs to be further studied.

Exercise interventions significantly decrease the severity of insomnia and alleviate sleep problems, according to the meta-analysis. The findings are consistent with those of previous studies (47, 48), which indicated the benefits of exercise intervention for insomnia in menopausal women. Exercise intervention usually consists of a set of activities that may have physiological and psychological benefits. The mechanism underlying the effectiveness of exercise on sleep outcomes has been proposed: exercises could increase energy expenditure, endorphin release, and body temperature in a way that enhances sleep for body recovery (49). Exercise reverses the sleep deprivation-induced decreased release of growth hormone (50). The benefits of exercise intervention for sleep problems have been confirmed in different populations (49, 51, 52).

For PSQI, the results showed that there were no significant differences between the exercise intervention group and the control group. A previous meta-analysis has found similar results (53). Nevertheless, another meta-analysis examined the efficacy of exercise training and found improved sleep quality in middle-aged and older adults with sleep problems (49), which is different from our findings. This may be because most included studies in our meta-analysis for synthesizing data of PSQI were conducted among menopausal women who had no sleep problems. Therefore, there are few opportunities for participants to improve the quality of their sleep. The results of subgroup analysis indicate that exercise intervention only had significant effects on sleep quality among menopausal women with sleep disorders rather than those without sleep disorders, which could offer evidence for the above explanation. Additionally, the subgroup analysis of individual with insomnia showed that more apparent effects of exercise intervention were found among women with sleep disorders than among women without sleep disorders. Hence, in further studies, it would be more appropriate to investigate the effects of exercise intervention in menopausal women who suffer from sleep disorders to understand the true effects of exercise.

Our results showed high heterogeneity, which may have resulted from the diverse exercise types. However, we did not conduct a more detailed classification for exercise types to carry out a quantitative analysis since each item had a limited number of studies. A previous meta-analysis observed that yoga did not have a significant reduction in PSQI scores compared to controls, while aerobic exercise had a favorable effect on sleep quality improvement (24). It is reported that meditative exercise were the most effective for sleep quality improvement (54). Therefore, more high-quality RCTs implementing different exercise types (e.g., walking, yoga, meditative exercise and so on) are needed to examine whether different intervention types may result in different effectiveness and enhance exercise interventions' clinical application.

It is worth noting that the exercise intervention duration ranged from 3 to 12 months. In our study, short-term exercise intervention appeared to be much more effective for PSQI score reduction than long-term exercise intervention among menopausal women. In contrast, better effects of long-term exercise intervention were found on insomnia compared to short-term intervention. The effects of exercise based on intervention duration (short-term or long-term) are still inconsistent in different populations (55–58). Which exercise intervention duration was more beneficial to sleep outcomes could not be judged. Therefore, more research needs to be done to provide implications for choosing an appropriate intervention duration in this field.

### 4.1. Strengths and limitations of the study

There is a growing concern over the health management of menopausal women (1, 3, 59). This study provides the latest evidence of the efficacy of exercise intervention on improving sleep in menopausal women and indicates some sleep management strategies for this population. The inclusion of RCTs in this study provided high standards for evidence-based research and was a strength of this study. Our research was conducted by two independent researchers and followed the PROSPERO statements as well as a prospective registered protocol, which ensured the rigor of this study.

There are some limitations in this study. First, significant publication bias was observed in regard to insomnia (*P* = 0.01), specifically suggesting that negative results may not have been published in articles. Future research is needed to explore this issue. Second, we did not perform subgroup analysis based on exercise types due to the limited number of studies. This may have led to significant heterogeneity in our study. Research shows that different types of exercise may lead to different intervention efficacies (24). Future studies should carry out further analysis based on exercise types to select the most effective exercise type to diminish sleep problems in menopausal women. Third, in all included studies, participants or personnel were not blinded, which increased the risk of bias. Consequently, caution should be taken when interpreting the findings of our study due to potential biases. Fourth, we did not examine the long-term effects of exercise intervention due to the limited number of studies and different assessment tools. Clinical trials with long-term follow-up intervals are needed to explore the long-term effectiveness of exercise intervention on sleep. Finally, most of the included studies only assessed subjective sleep using questionnaires such as the PSQI and ISI. The lack of objective sleep measures may influence the reliability of the results. Polysomnography (PSG) has been considered a standard tool for objectively quantifying sleep and evaluating most sleep disorders (60). Actigraphy is valid and reliable for assessing sleep patterns (61). These tools could be used to evaluate objective sleep in future studies.

## 5. Conclusion

Overall, the results of this meta-analysis of RCTs of exercise intervention for improving sleep among menopausal women indicated statistically significant effects on sleep-related outcomes. However, heterogeneity in this review warrants further exploration. The subgroup analysis revealed that the intervention duration and baseline severity of insomnia may influence the effectiveness of exercise. A future research agenda should conduct further analysis based on different study characteristics (e.g., baseline of participants, intervention types, duration and so on) to formulate appropriate intervention settings. Our results thus highlight that additional well-controlled RCTs applying different types of exercise (e.g., walking, yoga, meditative exercise and so on) with different intervention durations as well as subjective and objective sleep assessment should be performed on exercise intervention for sleep improvement in menopausal women to improve the rigor of the results found and to provide more sound clinical recommendations.

## Data availability statement

The original contributions presented in the study are included in the article/Supplementary material, further inquiries can be directed to the corresponding author.

## Author contributions

JQ and XY designed the study. JQ, SS, and MW participated in literature searches, study selection, and data extraction. YS and XS were responsible for checking data extraction as well as for the data analysis and statistical analysis. JQ and CJ participated in the writing of the manuscript. All authors have read and approved the submitted version.

## References

[1] Lobo RA Gompel A. Management of menopause: a view towards prevention. Lancet Diabetes Endocrinol. (2022) 10:457–70. 10.1016/S2213-8587(21)00269-2 35526556

[2] Kontis V Bennett JE Mathers CD Li G Foreman K Ezzati M. Future life expectancy in 35 industrialised countries: projections with a Bayesian model ensemble. Lancet. (2017) 389:1323–35. 10.1016/S0140-6736(16)32381-9 28236464 PMC5387671

[3] The LDE. Menopause: a turning point for women's health. Lancet Diabetes Endocrinol. (2022) 10:373. 10.1016/S2213-8587(22)00142-5 35526555

[4] Santoro N Roeca C Peters BA Neal-Perry G. The menopause transition: signs, symptoms, and management options. J Clin Endocrinol Metab. (2021) 106:1–15. 10.1210/clinem/dgaa764 33095879

[5] Kloss JD Perlis ML Zamzow JA Culnan EJ Gracia CR. Sleep, sleep disturbance, and fertility in women. Sleep Med Rev. (2015) 22:78–87. 10.1016/j.smrv.2014.10.005 25458772 PMC4402098

[6] Proserpio P Marra S Campana C Agostoni EC Palagini L Nobili L . Insomnia and menopause: a narrative review on mechanisms and treatments. Climacteric. (2020) 23:539–49. 10.1080/13697137.2020.1799973 32880197

[7] Baker FC Lampio L Saaresranta T Polo-Kantola P. Sleep and sleep disorders in the menopausal transition. Sleep Med Clin. (2018) 13:443–56. 10.1016/j.jsmc.2018.04.011 30098758 PMC6092036

[8] El KS Greendale G Crawford SL Avis NE Brooks MM Thurston RC . The menopause transition and women's health at midlife: a progress report from the study of Women's Health Across the Nation (SWAN). Menopause. (2019) 26:1213–27. 10.1097/GME.0000000000001424 31568098 PMC6784846

[9] Lee GB Kim HC Jung SJ. Association between sleep duration and augmentation index in post-menopausal women: a moderating role of depressive symptoms. Maturitas. (2021) 149:8–15. 10.1016/j.maturitas.2021.04.007 34134889

[10] Kim MJ Yim G Park HY. Vasomotor and physical menopausal symptoms are associated with sleep quality. PLoS ONE. (2018) 13:e192934. 10.1371/journal.pone.0192934 29462162 PMC5819793

[11] Chair SY Wang Q Cheng HY Lo SW Li XM Wong EM . Relationship between sleep quality and cardiovascular disease risk in Chinese post-menopausal women. BMC Womens Health. (2017) 17:79. 10.1186/s12905-017-0436-5 28893224 PMC5594540

[12] Baker FC de Zambotti M Colrain IM Bei B. Sleep problems during the menopausal transition: prevalence, impact, and management challenges. Nat Sci Sleep. (2018) 10:73–95. 10.2147/NSS.S125807 29445307 PMC5810528

[13] Rossouw JE Anderson GL Prentice RL LaCroix AZ Kooperberg C Stefanick ML . Risks and benefits of estrogen plus progestin in healthy postmenopausal women: principal results from the women's health initiative randomized controlled trial. JAMA. (2002) 288:321–33. 10.1001/jama.288.3.321 12117397

[14] Flores VA Pal L Manson JE. Hormone therapy in menopause: concepts, controversies, and approach to treatment. Endocr Rev. (2021) 42:720–52. 10.1210/endrev/bnab011 33858012

[15] Mehta J Kling JM Manson JE. Risks, benefits, and treatment modalities of menopausal hormone therapy: current concepts. Front Endocrinol. (2021) 12:564781. 10.3389/fendo.2021.564781 33841322 PMC8034540

[16] Yang CL Chen CH. Effectiveness of aerobic gymnastic exercise on stress, fatigue, and sleep quality during postpartum: a pilot randomized controlled trial. Int J Nurs Stud. (2018) 77:1–7. 10.1016/j.ijnurstu.2017.09.009 28950158

[17] Jurado-Fasoli L De-la-O A Molina-Hidalgo C Migueles JH Castillo MJ Amaro-Gahete FJ. Exercise training improves sleep quality: a randomized controlled trial. Eur J Clin Invest. (2020) 50:e13202. 10.1111/eci.13202 31989592

[18] Alkadhi KA. Exercise as a positive modulator of brain function. Mol Neurobiol. (2018) 55:3112–30. 10.1007/s12035-017-0516-4 28466271

[19] Mansikkamaki K Raitanen J Nygard CH Heinonen R Mikkola T EijaTomas S . Sleep quality and aerobic training among menopausal women: a randomized controlled trial. Maturitas. (2012) 72:339–45. 10.1016/j.maturitas.2012.05.003 22673453

[20] Tadayon M Abedi P Farshadbakht F. Impact of pedometer-based walking on menopausal women's sleep quality: a randomized controlled trial. Climacteric. (2016) 19:364–8. 10.3109/13697137.2015.1123240 26757356

[21] Aibar-Almazán A Hita-Contreras F Cruz-Díaz D de la Torre-Cruz M Jiménez-García JD Martínez-Amat A. Effects of pilates training on sleep quality, anxiety, depression and fatigue in postmenopausal women: a randomized controlled trial. Maturitas. (2019) 124:62–7. 10.1016/j.maturitas.2019.03.019 31097181

[22] Elavsky S McAuley E. Lack of perceived sleep improvement after 4-month structured exercise programs. Menopause. (2007) 14:535–40. 10.1097/01.gme.0000243568.70946.d4 17224851

[23] Aiello EJ Yasui Y Tworoger SS Ulrich CM Irwin ML Bowen D . Effect of a yearlong, moderate-intensity exercise intervention on the occurrence and severity of menopause symptoms in postmenopausal women. Menopause. (2004) 11:382–8. 10.1097/01.GME.0000113932.56832.27 15243275

[24] Rubio-Arias JÁ Marín-Cascales E Ramos-Campo DJ Hernandez AV Pérez-López FR. Effect of exercise on sleep quality and insomnia in middle-aged women: a systematic review and meta-analysis of randomized controlled trials. Maturitas. (2017) 100:49–56. 10.1016/j.maturitas.2017.04.003 28539176

[25] Berin E Hammar M Lindblom H Lindh-Åstrand L Spetz Holm AC. Effects of resistance training on quality of life in postmenopausal women with vasomotor symptoms. Climacteric. (2022) 25:264–70. 10.1080/13697137.2021.1941849 34240669

[26] Buchanan DT Landis CA Hohensee C Guthrie KA Otte JL Paudel M . Effects of yoga and aerobic exercise on actigraphic sleep parameters in menopausal women with hot flashes. J Clin Sleep Med. (2017) 13:11–8. 10.5664/jcsm.6376 27707450 PMC5181601

[27] Javadivala Z Allahverdipour H Asghari Jafarabadi M Emami A. An interventional strategy of physical activity promotion for reduction of menopause symptoms. Health Promot Perspect. (2020) 10:383–92. 10.34172/hpp.2020.57 33312934 PMC7722991

[28] Zhao J. Effects of Tai Chi Chuan on the changes of bone mineral density of perimenopausal women. Chin J Tissue Eng Res. (2020) 24:176–80. 10.3969/j.issn.2095-4344.190827216996

[29] Moher David Liberati Alessandro Tetzlaff Jennifer . Preferred reporting items for systematic reviews and meta-analyses: the PRISMA statement. BMJ. (2009) 339:332–6. 10.1136/bmj.b2535 21603045 PMC3090117

[30] Booth A Clarke M Dooley G Ghersi D Moher D Petticrew M . The nuts and bolts of PROSPERO: an international prospective register of systematic reviews. Syst Rev. (2012) 1:2. 10.1186/2046-4053-2-4 22587842 PMC3348673

[31] Buysse DJ Reynolds CR Monk TH Berman SR Kupfer DJ. The Pittsburgh Sleep Quality Index: a new instrument for psychiatric practice and research. Psychiatry Res. (1989) 28:193–213. 10.1016/0165-1781(89)90047-4 2748771

[32] Morin CM Belleville G Bélanger L Ivers H. The insomnia severity index: psychometric indicators to detect insomnia cases and evaluate treatment response. Sleep. (2011) 34:601–8. 10.1093/sleep/34.5.601 21532953 PMC3079939

[33] Wen J Li Y. The selection of a summary statistic for use in meta-analysis. Chin J Evid Based Med. (2007) 4:606–13.

[34] Li L Bing D Xinxin D. Effects of high-flow nasal cannuae in patients after extubation: a meta-analysis. Chin J Nurs. (2018) 53:1492–7. 10.3761/j.issn0254-1769.2018.12.01729149868

[35] Cohen J. Statistical Power Analysis for the Behavioral Sciences. Hillsdale, NJ: Baski (1988).

[36] Higgins JP Thompson SG Deeks JJ Altman DG. Measuring inconsistency in meta-analyses. BMJ. (2003) 327:557–60. 10.1136/bmj.327.7414.557 12958120 PMC192859

[37] Higgins JPT Thompson SG. Quantifying heterogeneity in a meta-analysis. Stat Med. (2002) 21:1539–58. 10.1002/sim.1186 12111919

[38] Higgins JP Altman DG Gotzsche PC Juni P Moher D Oxman AD . The cochrane collaboration's tool for assessing risk of bias in randomised trials. BMJ. (2011) 343:d5928. 10.1136/bmj.d5928 22008217 PMC3196245

[39] Abedi P Nikkhah P Najar S. Effect of pedometer-based walking on depression, anxiety and insomnia among postmenopausal women. Climacteric. (2015) 18:841–5. 10.3109/13697137.2015.1065246 26100101

[40] Wilbur J Miller AM McDevitt J Wang E Miller J. Menopausal status, moderate-intensity walking, and symptoms in midlife women. Res Theor Nurs Pract. (2005) 19:163–80. 10.1891/rtnp.19.2.163.66799 16025696

[41] Zhang J Chen G Lu W Yan X Zhu S Dai Y . Effects of physical exercise on health-related quality of life and blood lipids in perimenopausal women. Menopause. (2014) 21:1269–76. 10.1097/GME.0000000000000264 24937024

[42] Afonso RF Hachul H Kozasa EH de Souza Oliveira D Goto V Rodrigues D . Yoga decreases insomnia in postmenopausal women. Menopause. (2012) 19:186–93. 10.1097/gme.0b013e318228225f 22048261

[43] Newton KM Reed SD Guthrie KA Sherman KJ Booth-LaForce C Caan B . Efficacy of yoga for vasomotor symptoms. Menopause. (2014) 21:339–46. 10.1097/GME.0b013e31829e4baa 24045673 PMC3871975

[44] Luoto R Moilanen J Heinonen R Mikkola T Raitanen J Tomas E . Effect of aerobic training on hot flushes and quality of life—a randomized controlled trial. Ann Med. (2012) 44:616–26. 10.3109/07853890.2011.583674 21639722 PMC3469216

[45] Sternfeld B Guthrie KA Ensrud KE LaCroix AZ Larson JC Dunn AL . Efficacy of exercise for menopausal symptoms. Menopause. (2014) 21:330–8. 10.1097/GME.0b013e31829e4089 23899828 PMC3858421

[46] Kline CE Sui X Hall MH Youngstedt SD Blair SN Earnest CP . Dose–response effects of exercise training on the subjective sleep quality of postmenopausal women: exploratory analyses of a randomised controlled trial. BMJ Open. (2012) 2:e1044. 10.1136/bmjopen-2012-001044 22798253 PMC3400065

[47] Siu PM Yu AP Tam BT Chin EC Yu DS Chung KF . Effects of Tai Chi or exercise on sleep in older adults with insomnia: a randomized clinical trial. JAMA Netw Open. (2021) 4:e2037199. 10.1001/jamanetworkopen.2020.37199 33587135 PMC7885034

[48] Li S Li Z Wu Q Liu C Zhou Y Chen L . Effect of exercise intervention on primary insomnia: a meta-analysis. J Sports Med Phys Fitness. (2021) 61:857–66. 10.23736/S0022-4707.21.11443-4 34110122

[49] Yang PY Ho KH Chen HC Chien MY. Exercise training improves sleep quality in middle-aged and older adults with sleep problems: a systematic review. J Physiother. (2012) 58:157–63. 10.1016/S1836-9553(12)70106-6 22884182

[50] Ritsche K Nindl BC Wideman L. Exercise-induced growth hormone during acute sleep deprivation. Physiol Rep. (2014) 2:166. 10.14814/phy2.12166 25281616 PMC4254093

[51] Fang YY Hung CT Chan JC Huang SM Lee YH. Meta-analysis: Exercise intervention for sleep problems in cancer patients. Eur J Cancer Care. (2019) 28:e13131. 10.1111/ecc.13131 31353674

[52] Dolezal BA Neufeld EV Boland DM Martin JL Cooper CB. Interrelationship between sleep and exercise: a systematic review. Adv Prev Med. (2017) 2017:1364387. 10.1155/2017/1364387 28458924 PMC5385214

[53] Wang WL Chen KH Pan YC Yang SN Chan YY. The effect of yoga on sleep quality and insomnia in women with sleep problems: a systematic review and meta-analysis. BMC Psychiatry. (2020) 20:195. 10.1186/s12888-020-02566-4 32357858 PMC7193366

[54] Estévez-López F Maestre-Cascales C Russell D Álvarez-Gallardo IC Rodriguez-Ayllon M Hughes CM . Effectiveness of exercise on fatigue and sleep quality in fibromyalgia: a systematic review and meta-analysis of randomized trials. Arch Phys Med Rehabil. (2021) 102:752–61. 10.1016/j.apmr.2020.06.019 32721388

[55] Oudegeest-Sander MH Eijsvogels TH Verheggen RJ Poelkens F Hopman MT Jones H . Impact of physical fitness and daily energy expenditure on sleep efficiency in young and older humans. Gerontology. (2013) 59:8–16. 10.1159/000342213 22948012

[56] Chen KM Chen MH Lin MH Fan JT Lin HS Li CH. Effects of yoga on sleep quality and depression in elders in assisted living facilities. J Nurs Res. (2010) 18:53–61. 10.1097/JNR.0b013e3181ce5189 20220611

[57] Xie Y Liu S Chen XJ Yu HH Yang Y Wang W. Effects of exercise on sleep quality and insomnia in adults: a systematic review and meta-analysis of randomized controlled trials. Front Psychiatry. (2021) 12:664499. 10.3389/fpsyt.2021.664499 34163383 PMC8215288

[58] Burgess VN Antonio J Bland HW Wagner R Tartar JL Melton BF. The effect of timing and type of exercise on the quality of sleep in trained individuals. Int J Exerc Sci. (2020) 13:837–58. 32922649 10.70252/BKKE7434 PMC7449340

[59] Nappi RE Chedraui P Lambrinoudaki I Simoncini T. Menopause: a cardiometabolic transition. Lancet Diabet Endocrinol. (2022) 10:442–56. 10.1016/S2213-8587(22)00076-6 35525259

[60] Hirshkowitz M. Polysomnography and beyond. Principles Pract Sleep Med. (2017) 17:1564–6. 10.1016/B978-0-323-24288-2.00160-4

[61] Tsai SY Lee PL Gordon C Cayanan E Lee CN. Objective sleep efficiency but not subjective sleep quality is associated with longitudinal risk of depression in pregnant women: a prospective observational cohort study. Int J Nurs Stud. (2021) 120:103966. 10.1016/j.ijnurstu.2021.103966 34051587

