# Supplementary material for: The effect of exercise intervention on improving sleep in menopausal women: a systematic review and meta-analysis
Source: Front Med (Lausanne). 2023 Apr 25;10:1092294. doi: 10.3389/fmed.2023.1092294 (PMC10167708; doi:10.3389/fmed.2023.1092294)
Supplement: Supplementary file 1 [file Data_Sheet_1.docx]

**A: Subgroup analysis of sleep quality based on intervention population**

**
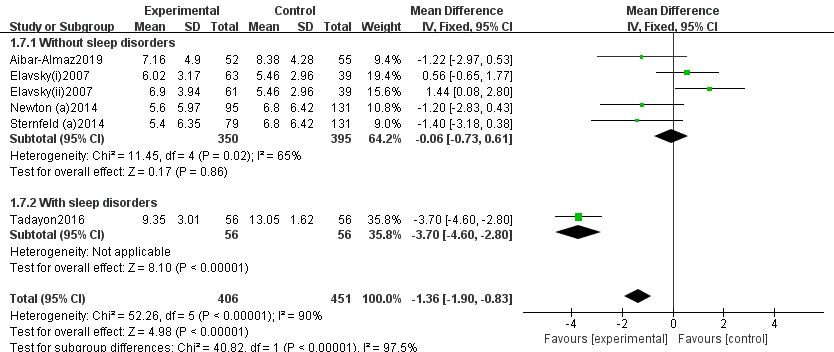
**

**B: Subgroup analysis of insomnia based on intervention population**

**
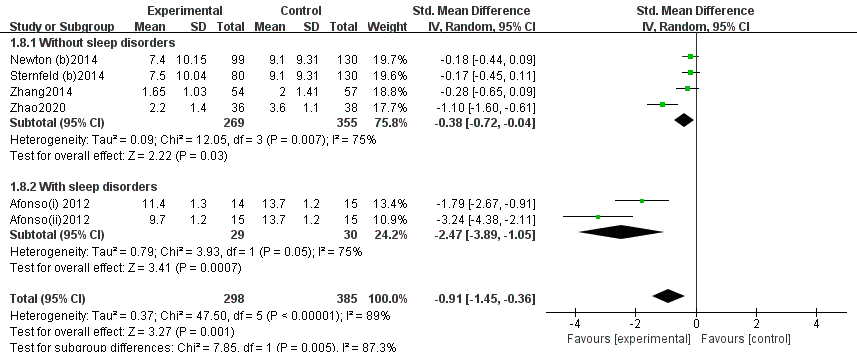
**

**Supplementary Figure 1.** Forest plots of the subgroup analyses: Without sleep disorders and with sleep disorders.
