# Supplementary material for: The effect of exercise intervention on improving sleep in menopausal women: a systematic review and meta-analysis
Source: Front Med (Lausanne). 2023 Apr 25;10:1092294. doi: 10.3389/fmed.2023.1092294 (PMC10167708; doi:10.3389/fmed.2023.1092294)
Supplement: Supplementary file 3 [file Table_1.DOCX]

**Supplementary Table 1 The final search strategies**

| Database | Search strategies | Results |
| --- | --- | --- |
| Cochrane Library | #1 MeSH descriptor: [Menopause] explode all trees  #2 MeSH descriptor: [Climacteric] explode all trees  #3 MeSH descriptor: [Perimenopause] explode all trees  #4 MeSH descriptor: [Postmenopause] explode all trees  #5 (menopause OR menopausal OR climacteric OR perimenopause OR perimenopausal OR peri-menopause OR peri-menopausal OR postmenopause OR postmenopausal OR post-menopause OR post-menopausal):ti,ab,kw (Word variations have been searched)  #6 #1 OR #2 OR #3 OR #4 OR #5  #7 MeSH descriptor: [exercise] explode all trees  #8 (exercis* OR physical activit* OR fitness OR aerobic training OR strength training OR resistance training):ti,ab,kw (Word variations have been searched)  #9 #7 OR #8  #10 MeSH descriptor: [Sleep] explode all trees  #11 MeSH descriptor: [Sleep Quality] explode all trees  #12 MeSH descriptor: [Sleep Initiation and Maintenance Disorders] explode all trees  #13 MeSH descriptor: [Sleep Wake Disorders] explode all trees  #14 (sleep OR sleep quality OR insomnia OR sleep disturbance* OR sleep maintenance OR sleeplessness OR sleep disorder* OR sleep problem*):ti,ab,kw (Word variations have been searched)  #15 #10 OR #11 OR #12 OR #13 OR #14  #16 ("randomized controlled trial"):pt (Word variations have been searched)  #17 (randomized):ti,ab,kw (Word variations have been searched)  #18 #16 OR #17  #19 #6 AND #9 AND #15 AND #18 | 150 |
| PubMed | 1. menopause[MeSH Terms] 2. climacteric[MeSH Terms] 3. perimenopause[MeSH Terms] 4. postmenopause[MeSH Terms] 5. menopause OR menopausal OR climacteric OR perimenopause OR perimenopausal OR peri-menopause OR peri-menopausal OR postmenopause OR postmenopausal OR post-menopause OR post-menopausal [Title/Abstract] 6. #1 OR #2 OR #3 OR #4 OR #5 7. aerobic exercise[MeSH Terms] 8. physical activity[MeSH Terms] 9. exercis* OR physical activit* OR fitness OR aerobic training OR strength training OR resistance training[Title/Abstract] 10. #7 OR #8 OR #9 11. sleeplessness [MeSH Terms] 12. sleep OR sleep quality OR insomnia OR sleep disturbance* OR sleep maintenance OR sleeplessness OR sleep disorder* OR sleep problem* 13. #11 OR #12 14. randomized controlled trial[Publication type] 15. randomized[Title/Abstract] 16. #14 OR #15 17. #6 AND #10 AND #13 AND #16 18. Filters applied: Randomized Controlled Trial | 49 |
| Embase | 1. 'menopause'/exp 2. 'climacterium'/exp 3. 'postmenopause'/exp 4. 'menopause'/exp OR 'menopause' OR 'menopausal' OR 'climacteric'/exp OR 'climacteric' OR 'perimenopause'/exp OR 'perimenopause' OR 'perimenopausal' OR 'peri-menopause' OR 'peri-menopausal' OR 'postmenopause'/exp OR 'postmenopause' OR 'postmenopausal' OR 'post-menopause'/exp OR 'post-menopause' 'post-menopausal':ab 5. #1 OR #2 OR #3 OR #4 6. 'exercise'/exp 7. 'physical activity'/exp 8. 'fitness'/exp 9. 'aerobic training'/exp 10. 'resistance training'/exp 11. 'exercis*' OR 'physical activit*' OR 'fitness'/exp OR 'fitness' OR 'aerobic training'/exp OR 'aerobic training' OR 'strength training'/exp OR 'strength training' OR 'resistance training':ab,ti 12. #6 OR #7 OR #8 OR #9 OR #10 OR #11 13. 'sleep'/exp 14. 'sleep quality'/exp 15. 'insomnia'/exp 16. 'sleep maintenance insomnia'/exp 17. 'sleep disorder'/exp 18. 'sleep'/exp OR 'sleep' OR 'sleep quality'/exp OR 'sleep quality' OR 'insomnia'/exp OR 'insomnia' OR 'sleep disturbance*' OR 'sleep maintenance' OR 'sleeplessness'/exp OR 'sleeplessness' OR 'sleep disorder*' OR 'sleep problem*':ab 19. #13 OR #14 OR #15 OR #16 OR #17 OR #18 20. 'randomized controlled trial':it 21. 'randomized':ab 22. #20 OR #21 23. #5 AND #12 AND #19 AND #22 24. #23 AND 'Article'/it | 125 |
| Web of Science | 1. menopause OR menopausal OR climacteric OR perimenopause OR perimenopausal OR peri-menopause OR peri-menopausal OR postmenopause OR postmenopausal OR post-menopause OR post-menopausal [Abstact] 2. exercis* OR physical activit* OR fitness OR aerobic training OR strength training OR resistance training [Abstact] 3. sleep OR sleep quality OR insomnia OR sleep disturbance* OR sleep maintenance OR sleeplessness OR sleep disorder* OR sleep problem* [Abstact] 4. randomized or RCT or random [Abstact] 5. 1 AND 2 AND 3 AND 4 6. Publication Type: CLINICAL TRIAL | 32 |
| PsycINFO | 1. menopause OR menopausal OR climacteric OR perimenopause OR perimenopausal OR peri-menopause OR peri-menopausal OR postmenopause OR postmenopausal OR post-menopause OR post-menopausal [Abstact] 2. exercis* OR physical activit* OR fitness OR aerobic training OR strength training OR resistance training [Abstact] 3. sleep OR sleep quality OR insomnia OR sleep disturbance* OR sleep maintenance OR sleeplessness OR sleep disorder* OR sleep problem* [Abstact] 4. randomized or RCT or random [Abstact] 5. 1 AND 2 AND 3 AND 4 | 13 |
| CINAHL | 1. menopause OR menopausal OR climacteric OR perimenopause OR perimenopausal OR peri-menopause OR peri-menopausal OR postmenopause OR postmenopausal OR post-menopause OR post-menopausal [Abstact] 2. exercis* OR physical activit* OR fitness OR aerobic training OR strength training OR resistance training [Abstact] 3. sleep OR sleep quality OR insomnia OR sleep disturbance* OR sleep maintenance OR sleeplessness OR sleep disorder* OR sleep problem* [Abstact] 4. randomized or RCT or random [Abstact] 5. 1 AND 2 AND 3 AND 4 | 26 |
| ProQuest | 1. menopause OR menopausal OR climacteric OR perimenopause OR perimenopausal OR peri-menopause OR peri-menopausal OR postmenopause OR postmenopausal OR post-menopause OR post-menopausal [Abstact] 2. exercis* OR physical activit* OR fitness OR aerobic training OR strength training OR resistance training [Abstact] 3. sleep OR sleep quality OR insomnia OR sleep disturbance* OR sleep maintenance OR sleeplessness OR sleep disorder* OR sleep problem* [Abstact] 4. randomized or RCT or random [Abstact] 5. 1 AND 2 AND 3 AND 4 | 32 |
| Scopus | ( TITLE ( menopause OR menopausal OR climacteric OR perimenopause OR perimenopausal OR peri-menopause OR peri-menopausal OR postmenopause OR postmenopausal OR post-menopause OR post-menopausal ) ) AND ( TITLE-ABS-KEY ( exercis* OR "physical activit*" OR fitness OR "aerobic training" OR "strength training" OR "resistance training" ) ) AND ( TITLE-ABS-KEY ( sleep OR "sleep quality" OR insomnia OR "sleep disturbance*" OR "sleep maintenance" OR sleeplessness OR "sleep disorder*" OR "sleep problem*" ) ) AND ( TITLE-ABS-KEY ( randomized OR rct OR random ) ) AND ( LIMIT-TO ( DOCTYPE , "ar" ) ) | 117 |
