# Supplementary material for: The effect of exercise intervention on improving sleep in menopausal women: a systematic review and meta-analysis
Source: Front Med (Lausanne). 2023 Apr 25;10:1092294. doi: 10.3389/fmed.2023.1092294 (PMC10167708; doi:10.3389/fmed.2023.1092294)
Supplement: Supplementary file 4 [file Table_2.docx]

**Supplementary Table 2 Study characteristics (n=17)**

| **First author**  **(publication year)** | **Country** | **Population** | **Sample size (intervention/control)** | **Type of**  **exercise** | **Intervention duration, frequency, and session** | **Control** | **Evaluation time points** | **Assessment tools** |
| --- | --- | --- | --- | --- | --- | --- | --- | --- |
| ^a^Abedi et al. (2015) | Iran | Menopausal women with mild to moderate depression | 49/48 | Pedometer-based walking | 12 weeks walking; women were advised to increase their steps by at least 500 steps per week. | Usual care | Baseline, 4th, 8th, and 12th weeks of intervention | GHQ-28 |
| Afonso et al.  (2012) | Brazil | Menopausal women with insomnia | 14/15/15 | Passive stretching/Yoga | Passive stretching group: two 1-hour sessions per week lasting 4 months; participants would lie on a stretcher, first on her back and then on her stomach, and the main articulations in her body would be manipulated, with a soft stretching of the main muscles of those articulations.  Yoga group: participants completed two sessions a week that lasted 1 hour each lasting 4 months; the yoga uses stretching positions along with strong and fast breathing. The practice ended with a directed relaxation. | Usual care | Preintervention and postintervention | ISI |
| Aibar-Almazan et al.  (2019) | Spain | Postmenopausal women | 52/55 | Pilates training | Participants in the intervention group attended two one-hour sessions of Pilates exercises each week for 12 weeks. Each Pilates training session included warm-up (10 min), main Pilates training activity (35 min), and cool-down (15 min). | Usual care | Preintervention and postintervention | PSQI |
| ^a^Aiello et al.  (2004) | USA | Postmenopausal women aged 50-75 | 87/86 | Moderate-intensity exercise including walking, aerobics, and bicycling | The exercise intervention consisted of at least 45 minutes of moderate-intensity exercise, 5 days per week (goal of 225 min per week) for 12 months. | Women in the control group were asked not to change other exercise habits during the study. | Baseline, 3-, 6-, 9-, and 12-month follow-up measures | Participants were asked if they experienced trouble sleeping or awakening too early |
| ^a^Berin et al.  (2022) | Sweden | Postmenopausal women >45 years old with daily vasomotor symptoms | 27/29 | Resistance training | 15-week resistance training program; participants were instructed to train three times per week, with one session per week | Usual Activity | Preintervention and postintervention | WHQ |
| ^a^Buchanan et al.  (2017) | USA | Late transition and postmenopausal women aged 40–62 y with hot flashes | 52/54/80 | Yoga/aerobic exercise | 1. week yoga: the yoga intervention was based on the Viniyoga style. The 12 weekly classes were 90 min long (attended once weekly). The participants were instructed to practice at home for 20 min daily on nonclass days, alternating between poses one day and Yoga Nidra the next day.   12-week aerobic exercise: Participants in the aerobic exercise group attended 12 weeks of in-person training, with three weekly sessions. The sessions typically lasted 40-60 min. | Usual activity | Preintervention and postintervention | Actigraphic Sleep Outcomes |
| Elavsky et al.  (2007) | USA | Middle-aged women (42-58 years) experiencing vasomotor symptoms | 63/61/39 | Walking/Yoga | The 4-month walking program involved moderate-intensity exercise three times per week for 1 hour. The duration started at 15 minutes of sustained exercise (i.e., after warm-up) and gradually increased to 40 to 45 minutes by the midpoint of the exercise program.  The 4-month yoga program was of low intensity, meeting twice per week for 90 minutes. Iyengar yoga was practised with a focus on developing strength, stamina, flexibility, and balance, as well as concentration and meditation. | No treatment | Preintervention and postintervention | PSQI |
| ^a^Javadivala et al.  (2020) | Iran | Menopausal and perimenopausal women aged 40-60 | 93/68 | Physical activities including walking, stretching and vibration exercise | 12-week regular physical activity program: the program started at a half-hour daily of low-level activity, progressing to an hour of moderate level activity by the final week | No treatment | Preintervention and postintervention | MSR |
| ^a^ Kline et al.  (2012) | USA | Postmenopausal women aged  45-75 years | 155/104/103/102 | Semirecumbent cycle ergometer and treadmill exercise | Women in the exercise groups participated in three to four training sessions/week for 6 months. Exercise dosages were structured to elicit energy expenditures of 4, 8 or 12 KKW, respectively. | No treatment | Preintervention and postintervention | MOS Sleep Scale |
| Luoto et al.  (2012) | Finland | Women aged 43-63 years with  menopausal symptoms | 74/80 | Aerobic training | Six-month unsupervised aerobic training intervention: the exercise programme included aerobic training 4 times per week, with 50 min of exercise each time. | No treatment | Preintervention and postintervention | WHQ |
| ^a^Mansikkamäki et al.  (2012) | Finland | Same as above | 73/76 | Aerobic training | Same as above | No treatment | Twice a day during the trial | Two questions  on sleep quality (yes/no) |
| Newton et al.  (2014) | USA | Women aged 40-62 years in the menopausal transition or postmenopausal | 107/142 | Yoga | 12-week yoga: yoga instruction was provided during 12 weekly 90-minute classes. Classes were offered twice weekly. Participants were instructed to practice at home 20 minutes each day they did not attend class. | Usual routine | At baseline, 6 weeks and 12 weeks | ISI, PSQI |
| Sternfeld et al.  (2014) | USA | Women aged 40-62 years in the menopausal transition or postmenopausal | 106/142 | Aerobic training | 12-week exercise: 3 sessions per week; Women chose whether to exercise on a treadmill, elliptical trainer, or stationary bicycle. Duration typically ranged from 40-60 minutes/session. | Usual routine | At baseline, 6 weeks and 12 weeks | ISI, PSQI |
| Tadayon et al.  (2016) | Iran | Postmenopausal  Women with sleep disorders | 56/56 | Pedometer-based walking | Participants were asked to start by walking distances comfortable for them, and then to add 500 steps each week to reach a maximum of 10000 steps per day by the end of 12 weeks. | No treatment | At baseline, 4 weeks, 8 weeks, and 12 weeks after intervention. | PSQI |
| ^a^Wilbur et al.  (2015) | USA | Midlife women aged 45-65 years | 97/66 | Walking | 24-week, home-based, moderate-intensity walking intervention: the exercise prescription was walking at a frequency of 4 times a week for a duration of 20-30 minutes. | No treatment | At baseline and 24 weeks | Symptom Impact Inventory-Sleep |
| Zhang et al.  (2014) | China | Female medical staff aged 40 to 55 years and with a Kupperman index score of 15 points or higher | 54/57 | Walking | 12-week walking program: the intervention group was required to exercise three times a week or more, with each session lasting at least 30 minutes. | No treatment | At baseline, 4 weeks, 8 weeks and 12 weeks | KMI-Insomnia |
| Zhao et al.  (2020) | China | Perimenopausal women aged 45-55 years | 36/38 | Tai Chi Chuan | 48-week Tai Chi Chuan exercise: 3 times a week, with each session lasting 60 minutes. | No treatment | Preintervention and postintervention | KMI-Insomnia |

^a^ Not included in the meta-analysis

Abbreviations: GHQ: General Health Questionnaire; ISI: Insomnia Severity Index; PSQI: Pittsburgh Sleep Quality Index; WHQ: Women’s Health Questionnaire; SIMW: Sleep Index for Midlife Women; MSR: Menopause Rating Scale; MOS: Medical Outcomes Study; KKW: kilocalories per kilogram of body weight per week; KMI: Kupperman index.
